# Anatomical Response and Infection of Soybean during Latent and Pathogenic Infection by Type A and B of *Phialophora gregata*

**DOI:** 10.1371/journal.pone.0098311

**Published:** 2014-05-30

**Authors:** Ann E. Impullitti, Dean K. Malvick

**Affiliations:** Department of Plant Pathology, University of Minnesota, St. Paul, Minnesota, United States of America; Nanjing Agricultural University, China

## Abstract

Growth and anatomical responses of plants during latent and pathogenic infection by fungal pathogens are not well understood. The interactions between soybean (*Glycine max*) and two types of the pathogen *Phialophora gregata* were investigated to determine how plants respond during latent and pathogenic infection. Stems of soybean cultivars with different or no genes for resistance to infection by *P. gregata* were inoculated with wildtype or GFP and RFP-labeled strains of types A or B of *P. gregata*. Plants were sectioned during latent and pathogenic infection, examined with transmitted light or fluorescent microscopy, and quantitative differences in vessels and qualitative differences in infection were assessed using captured images. During latent infection, the number of vessels was similar in resistant and susceptible plants infected with type A or B compared to the control, and fungal infection was rarely observed in vessels. During pathogenic infection, the resistant cultivars had 20 to 25% more vessels than the uninfected plants, and fungal hyphae were readily observed in the vessels. Furthermore, during the pathogenic phase in a resistant cultivar, *P.gregata* type A-GFP was limited to outside of the primary xylem, while *P.gregata* type B-RFP was observed in the primary xylem. The opposite occurred with the susceptible cultivar, where PgA-GFP was observed in the primary xylem and PgB-RFP was limited to the interfascicular region. In summary, soybean cultivars with resistance to BSR produced more vessels and can restrict or exclude *P. gregata* from the vascular system compared to susceptible cultivars. Structural resistance mechanisms potentially compensate for loss of vessel function and disrupted water movement.

## Introduction

Symbiosis between plants and fungi can take on many forms, such as mutualistic, pathogenic, or commensal. These interactions are well-defined in many organisms, but these interactions can be complicated and can sometimes transition between forms [1], [2], [3], [4]. For example during pathogenesis, some fungal pathogens lead a latent lifestyle in which the plant and pathogen initially co-exist for some period of time without any apparent symptoms of disease and for unknown reasons disease and symptom development occurs. It is unknown why pathogens lead latent lifestyles, but is hypothesized to be due to an unfavorable environment for disease development that later change to favorable, and also the possibility of greater susceptibility to disease as the plant matures and the environment changes. Latent lifestyles differ from endophytic lifestyles, as by definition endophytes do not cause symptoms of disease throughout the association [5].

Several studies have investigated how fungi differentially colonize their hosts during asymptomatic and symptomatic infection. During asymptomatic infection (latent or endophytic) fungal hyphae often colonize plant tissues intercellularly, while pathogenic infection is characterized by intracellular infection [5], [6], [7], [8]. *Fusarium verticilliodes* infects corn and has been referred to as an endophyte, latent pathogen, and pathogen depending on the environment, water availability, and genetics of the host and fungus [6], [7], [9], [10], [11]. During asymptomatic infection, hyphae of *F. verticilliodes* only colonize intercellular spaces, but during symptomatic infection, hyphae are observed in stem tissues inter- and intracellularly, and stalk biomass is reduced [6], [7], [10]. In another example, pathogenic infection of strawberry and endophytic infection of solanaceous hosts by *Colletotrichum acutatum* were investigated [8]. On strawberry, a penetration peg infected host tissue, hyphae colonized intracellularly, and necrosis developed. On solanaceous hosts, appresoria of *C. acutatum* either did not penetrate the cuticle, the fungus had an epiphytic lifestyle, or hyphae were restricted to intercellular growth [8]. These investigations have revealed differences in localized infection and colonization with asymptomatic and pathogenic interactions, but much remains to be understood about fungal infection within hosts and how plants respond to the infection.

The primary anatomical response of plants to fungal infections is induced structural defenses, such as cell wall thickening or papillae, that prevent pathogens from penetrating host cells. There is also some evidence suggesting that the development of the vascular structure of plants can be disrupted during pathogen infection, and the cambium layer reduced in infected plants compared to uninfected plants [12], [13]. Research on how the vascular anatomy of plants is modified due to infection by vascular pathogens is scarce. Tomato plants infected by a vascular pathogen have fewer vessels during infection, but the relationship between vessel size and infection is less clear [12].

The interactions between soybean (*Glycine max* (L.) Merr.) and *Phialophora gregata* W.Gams (Allington and Chamberlain) [*Cadophora gregata* Harrington and McNew [14]], a common soybean infecting fungus that causes the yield reducing disease brown stem rot (BSR) were used to investigate latent and pathogenic infection. *P. gregata* is a soybean pathogen that infects plants during seedling stages [15]. Infection in field-gown plants is latent for 8 to 12 weeks and then transitions to pathogenic with symptoms of disease developing on leaves and/or in stems 10 to 14 weeks after planting during reproductive growth stages [15], [16]. Symptoms of BSR, which include leaf abscission, interveinal chlorosis and necrosis of leaves, and internal browning of vascular and pith tissues are caused by two different types of the pathogen [17], [18]. *P. gregata* is a unique pathogen in that the two types are genetically distinct based on sequence variation in the nuclear IGS region of rDNA and the ability (type A) or inability (type B) to cause severe interveinal chlorosis and necrosis on soybean leaves [19], [20].

Latent pathogenesis and the transition to the disease state with symptom development on leaves and/or stems are not well understood in plants. This study focuses on what is happening to the plant and the fungus in soybean plants during these two very different stages. The primary aim of this study was to determine if the internal anatomy [21] of soybean is modified due to differences in latent and pathogenic infection, and to investigate how two genetically distinct types of the same pathogen interact within plants. There have been very few previous investigations on the anatomical response of plants due to latent infection. The objectives of this study were to: 1) Compare the vascular anatomy of stems of resistant and susceptible soybean cultivars at different time points following infection with types A and B of *P. gregata*; 2) determine if types A and B of *P. gregata* colonize resistant and susceptible soybean stems differently during latent and pathogenic infection; 3) Determine if simultaneous infection of plants by types A and B results in concurrent infection of the same plant tissues. These studies improve the understanding of colonization of plant tissues by revealing how two types of the same pathogen colonize and interact *in planta*, and how susceptible and partially resistant cultivars respond to infection by altering their anatomies during latent and pathogenic infection.

## Materials and Methods

### Isolate descriptions and transformation

The type A isolate (OH2-3) of *P. gregata*, transformed to express GFP (PgA-GFP) was supplied by C. Bronson at Iowa State University, and the type B isolate (MNRF-ss1) of *P. gregata* was transformed at the University of Minnesota to express RFP using a protocol provided by Iowa State University. A single spore isolate was grown on green bean agar (GBA, [22]) for 4 weeks, and then 5 ml of a conidial suspension was incubated in darkness at 25°C for 48 hrs to promote germination. Protoplasts were prepared from the germinating conidia using a solution containing 7.5 mg/ml driselase, 100 µg/ml chitinase, and 7.5 mg/ml Glucanex (Sigma-Aldrich, St. Louis, MO) [23], [24]. The RFP vector, pCA56, supplied by L. Ciuffetti at Oregon State University, was linearized with *Sac*I prior to polyethylene glycol (PEG) transformation of the protoplasts [23], [24], [25], [26]. The protoplasts were mixed into a regeneration medium and 15 ml were poured into petri plates [23]. Cell walls were regenerated for 24 hrs at 24°C, and then the plates were overlaid with 1% water agar containing 150 µg/ml hygromycin B and incubated for 14 days at 25°C. Potential transformants were transferred to GBA containing 100 µg/ml hygromycin B. RFP isolates were stored in sterile water at 14°C.

Transformed isolates were characterized and compared to wild-types (WT) strains. Insertion of RFP into the type B isolate was confirmed by PCR with the primers U32 and U33, and expression of RFP was verified by fluorescent microscopy [27]. Only the transformants had a pink phenotype on culture-media [27]. Two RFP transformants with morphology similar to the WT were selected for phenotypic comparison with the WT. Growth was assessed by placing 5 mm diameter disks of actively growing cultures on GBA, incubating at 25°C with a 12 hr photoperiod for 3 weeks, and then measuring the colony diameters. Sporulation and dry mass were assessed by growing the WT and transgenic isolates in soybean seed broth (SSB) (12 g dry soybean seed/100 ml water) at 23°C with ambient room fluorescent light for 21 days. Then, conidia were enumerated using a hemacytometer, and mycelium biomass was determined by collecting mycelium using filtration, followed by drying at 35°C for 12 hrs, and then weighing. Experiments comparing the growth of the wild-type and transformed isolates were replicated three times.

Virulence and pathogenicity of transformed and WT isolates were assessed by growing isolates type A-WT (OH2-3), type B-WT (MNRF-ss1), type A-GFP (PgA-GFP), and type B-RFP (PgB-RFP) in SSB for 21 days. Soybean cultivars Bell and Corsoy 79 were grown in vermiculite to the VC-V1 growth stage and inoculated by dipping wounded roots into a suspension of 6.0×0^6^ cfu/ml conidia [20]. Three inoculated plants per treatment were transplanted into 15.2 cm pots containing Sunshine LP5 (Sun Gro Horticulture, Bellevue, WA) and grown in a growth chamber with a 12 hr photoperiod at a 23°C day and 20°C night temperature for 7 weeks. Percent foliar disease severity was based on the number of healthy and symptomatic trifoliates, and stem symptom severity was determined by splitting stems to assess necrosis. Experiments comparing the virulence and pathogenicity of the wild-type and transformed isolates were replicated three times.

### Evaluation of soybean anatomy and infection phases following inoculation with WT isolates

Two experiments (1 and 2) were conducted using the susceptible cultivar Corsoy 79 and the resistant cultivar, Bell [28], [29], and the third experiment used the resistant cultivars, IA2008R (*Rbs3*) [30], the near isogenic resistant line LN 92-12033 (*Rbs2*), and its corresponding susceptible line LN 92-12054 [28], [31], [32], [33]. The resistance to BSR noted here has likely been characterized primarily with type A, which may or may not correlate to resistance to type B. Plants were inoculated with WT isolates and grown in the growth chamber as described above.

Stem segments taken from the base (at the soil-line), middle, and apex of each plant 1, 2, 4, 6, and 8 weeks post-inoculation (WPI) were embedded in 6% water agar, and 5 to 20 µm thick cross-sections (XS) were obtained with a Vibratome 1000 Plus Sectioning System (Vibratome Co., St. Louis, MO). Sections were mounted on a microscope slide, stained with lactophenol aniline blue (Remel, Lenexa, KS), washed with water, and examined with an Eclipse e600 Nikon Microscope (Nikon Instruments Inc., Melville, NY). Transmitted light microscopy (TLM) images were captured using a Nikon DXMF 1200 camera with the software ACT1. Complete cross sections of the base and apex could not be obtained due to secondary and immature tissues, respectively, and these sections were used only to determine the presence or absence of *P. gregata*. A composite picture of the middle stem cross-section was taken at 40x, and continuous images of the entire stem cross-section were captured at 200x. The number of vessels, area of vessels, and percent of vessels colonized with *P. gregata* in the middle section of stems were determined by analyzing images using Image J software (National Institutes of Health [34]. For experiment 1, sections from weeks 1, 2, 4, 6, and 8 WPI were analyzed. Based on those results, data in experiments 2 and 3 were collected only at 2 and 6 WPI for analysis of latent and pathogenic infection, respectively. Longitudinal sections (LS) of stems were collected at 2 WPI, and root XS and LS were taken at 2 and 8 WPI. A total of 55,113 vessels (n = 168 per middle stem XS) were analyzed from the transmitted light experiments. The stem segments adjacent to the area used for analysis of cross-sections were also collected during each experiment, chopped into 1 to 2 mm pieces, and stored at −20°C prior to extraction of DNA and analysis with standard PCR (sPCR) and more sensitive real-time quantitative PCR (qPCR) to confirm presence of *P. gregata* [19], [35].

### Assessment of infection by types A and B using fluorescent isolates

Cultivars Bell and Corsoy 79 were inoculated at the V1 growth stage as described with isolates PgA-GFP and PgB-RFP separately and with 1∶1 mixture of conidia of both isolates. Inoculated and non-inoculated plants were grown, symptoms assessed, and plant tissues sectioned as described. A Nikon C1si laser scanning confocal microscope (LSCM) at the University of Minnesota Imaging Center was used for fluorescent microscopic analysis, which allows unmixing of autofluorescence from the plant and fluorescence from GFP and RFP isolates. Images of the GFP isolate were acquired by excitation with a 488 argon laser at 12% intensity in standby modus, and images of the RFP isolate were acquired following excitation at 561 nm at 15% intensity, with the fluorescence being detected with 32-channel photo multiplier tubes (PMTs) at a range of 505 to 680 nm which were corrected for differential wavelength sensitivity. A spectral gain of 160 and pixel dwell speed of 6 µs was used. The images were projections of a z-series (maximum intensity projection images) collected at 0.5 micron steps. The z-series merger and unmixing of autofluorescence and GFP and/or RFP fluorescence was done using known GFP and RFP spectra (Clontech, Mountain View, CA) and by selecting regions of auto-fluorescence in control plants using the Nikon EZ-C1 Viewer (v3.60).

### Statistical analysis

Statistical analyses were conducted using GraphPad Prism (Graphpad v6.02, GraphPad Software Inc., La Jolla, CA). The data from the three similar experiments that evaluated transformants were not significantly different and were combined for analysis using ANOVA, and then means were compared using a Fisher's LSD (P = 0.05). Data from the transmitted light experiments that assessed the quantity of vessels and average area of vessels were analyzed separately by an ANOVA, followed by a Fisher's LSD.

## Results

### Evaluation of transformed isolates

Several conidia of *P. gregata* type B were transformed to express RFP, and one of the transformants (named ‘PgB-RFP’) had a similar phenotype, as well as radial growth rate, dry mycelial mass, and sporulation statistically the same as the WT isolate (Table 1). Likewise, after inoculation, the stem symptom severity of the resistant cultivar Bell, and the susceptible cultivar, Corsoy 79, were similar when inoculated with either the WT or PgB-RFP isolate (Table 1). As expected and similar to other WT PgB isolates, few to no foliar symptoms were observed following inoculation with this isolate. The expression of RFP was stable and bright throughout the experiments, and no loss of fluorescence or photo bleaching after LSCM was observed.

**Table 1:** Comparison of growth and virulence of wild-type and transformed isolates of *Phialophora gregata* Type B.

				Stem Severity (%)^c^	
Isolate	Colony Diameter (cm)^a^	Dry Mass (mg)^b^	Conidia/ml^b^	Bell^d^	Corsoy 79^e^
MNRF-ss1^f^	3.43	31.8	9.6×10^5^	16	30
MNRF-ss1-RFP^g^	3.62	30.38	8.2×10^5^	13	24
MNRF-ss1-RFP2	1.32	24.3	6.8×10^4^	0	5
P value^h^	<0.01	<0.01	<0.01	<0.01	<0.01
LSD (p = 0.05)	0.72	5.5	6.5×10^5^	6	6

a Measurements taken after 21 days of growth on green bean agar.

b Measurements taken after 21 days of growth in soybean seed broth.

c Stem ratings are the mean of six stems 7 weeks after inoculation.

d Resistant cultivar.

e Susceptible cultivar.

f Wild-type (WT).

g Red Fluorescent Protein (RFP).

h Experiments were replicated three times and data were combined for statistical analysis (n = 9).

### Colonization of the vascular system during latent and pathogenic infection

**Figure 1:**
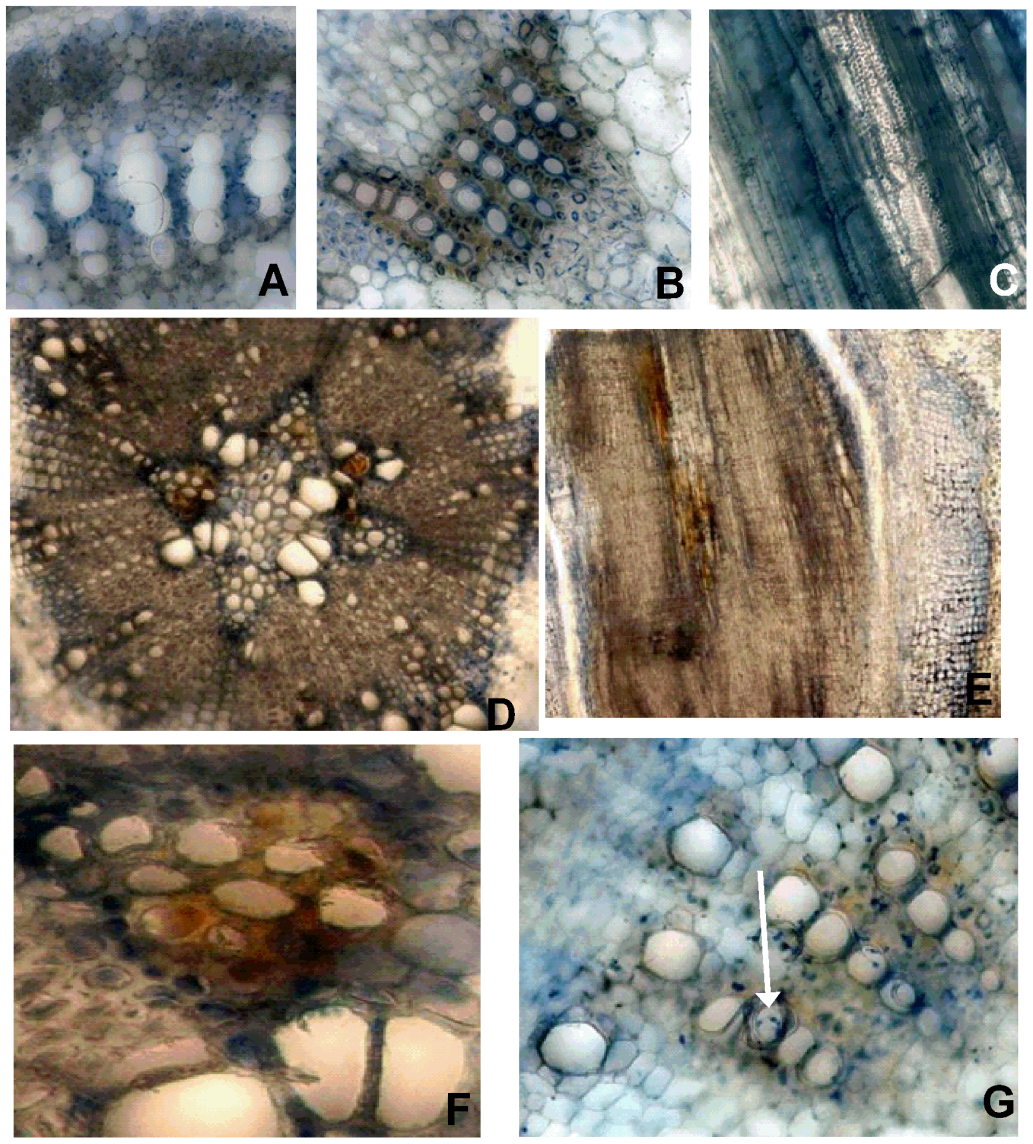
Latent infection in soybean stems infected with *Phialophora gregata*. Latent infection as observed in stem cross sections from a BSR-susceptible soybean cultivar (Corsoy 79) infected with wild-type isolates of types A and B of *Phialophora gregata*. Images A–F were captured 2 weeks post inoculation. (A) No infection was observed in the cross sections of the apex (B) or the cross section and (C) longitudinal section of the middle of the non-inoculated control plant. (D) Necrosis in the vascular system of the root, (E) longitudinal section of a root, (F) a necrotic region from D that was cropped and magnified. Image G was captured 3 weeks post inoculation and hyphae) are beginning to colonize the xylem vessels (noted by arrow). Image A is 40x magnification, B, C, and E–G are 200x magnification, D is 100x magnification.

At 1 WPI, *P. gregata* was not detected using sPCR or qPCR, no evidence of fungal infection was observed in the root, base, middle, or apex sections of infected plants, and no visible symptoms of infection developed (data not shown). At 2 WPI no visible foliar or stem symptoms were observed in resistant or susceptible cultivars inoculated with *P. gregata* (Table 2). At that time, however, PgA and PgB were detected in stem tissue of inoculated susceptible cultivars using sPCR and qPCR, but were not detected in non-inoculated plants. Differences in infection of root and stem tissues were also observed 2 WPI. In the roots, necrosis was observed in the interfascicular region and *P. gregata* was observed in the vascular system (Figure 1d–f). In the stem, *P. gregata* was limited to the interfascicular areas, and less than 10% of xylem vessels were colonized (Fig. 1a–c and Table 2). At 4 WPI, types A and B of *P. gregata* were observed in the xylem vessels of the base, middle, and apex of resistant and susceptible plants using TLM, but neither type A or type B was observed in the pith (parenchyma) or cortex of stems and visible symptoms of disease were not present (data not shown).

**Table 2:** Detection of *Phialophora gregata* in stems with PCR, observation of hyphae in vessels, and stem symptom severity of resistant and susceptible cultivars at 2 and 6 weeks post inoculation.

		2 Weeks Post Inoculation				6 Weeks Post Inoculation		
Cultivar and Inoculation	No. of vessels assayed	Detection of *P. gregata* (%)^a^	Vessels Infected (%)^b^	Stem Severity (%)	No. of vessels assayed	Detection of *P. gregata* (%)	Vessels Infected (%)	Stem Severity (%)
Bell (R)^c^								
Expt 1^f^								
A^d^	574	100	6.2	0	1074	100	6.9	13
B^e^	977	33	6.1	0	717	100	13.9	2
Control	769	0	1.2	0	786	0	0.0	0
Expt. 2^f^								
A	870	100	1.1	0	816	100	3.0	10
B	956	33	1.6	0	698	100	29.9	2
Control	920	0	0.0	0	589	0	0.0	0
Corsoy 79 (S)								
Expt. 1								
A	704	0	8.8	0	714	100	49.2	45
B	727	100	3.2	0	734	100	17.8	12
Control	692	0	0.7	0	806	0	0.4	0
Expt. 2								
A	730	100	1.3	0	736	100	65.4	73
B	779	33	3.2	0	748	100	14.1	7
Control^g^	617	0	0.2	0	1023	0	0.0	0
IA 2008 (R)								
A^h^	750	33	1.3	0	1586	50	0.8	10
B	929	0	0.6	0	1252	100	5.7	23
Control	795	0	0.1	0	1196	0	0.0	0
LN 92-12033 (R)								
A	730	0	0.0	0	1812	75	7.9	6
B	821	50	0.1	0	990	100	27.7	50
Control	906	0	0.0	0	1502	0	0.0	0
LN 92-12054 (S)								
A	673	75	0.0	0	807	100	73.3	50
B	616	100	0.0	0	1224	100	25.2	60
Control	634	0	0.0	0	1698	0	0.0	0

a Detection of *P. gregata* in plants. If *P. gregata* was not detected using standard PCR, a more sensitive real-time quantitative PCR assay was used.

b The average of the total number of vessels infected with *P. gregata* in a cross section.

c Reaction of soybean cultivars to *P. gregata*; R =  resistant, S =  susceptible.

d Plants inoculated with Type A of *P. gregata*.

e Plants inoculated with Type B of *P. gregata*.

f Treatments were replicated three times (n = 3 plants/replication).

g Treatments were replicated three times (n = 2 plants/replication).

h Treatments were replicated three times (n = 4 plants/replication).

**Figure 2:**
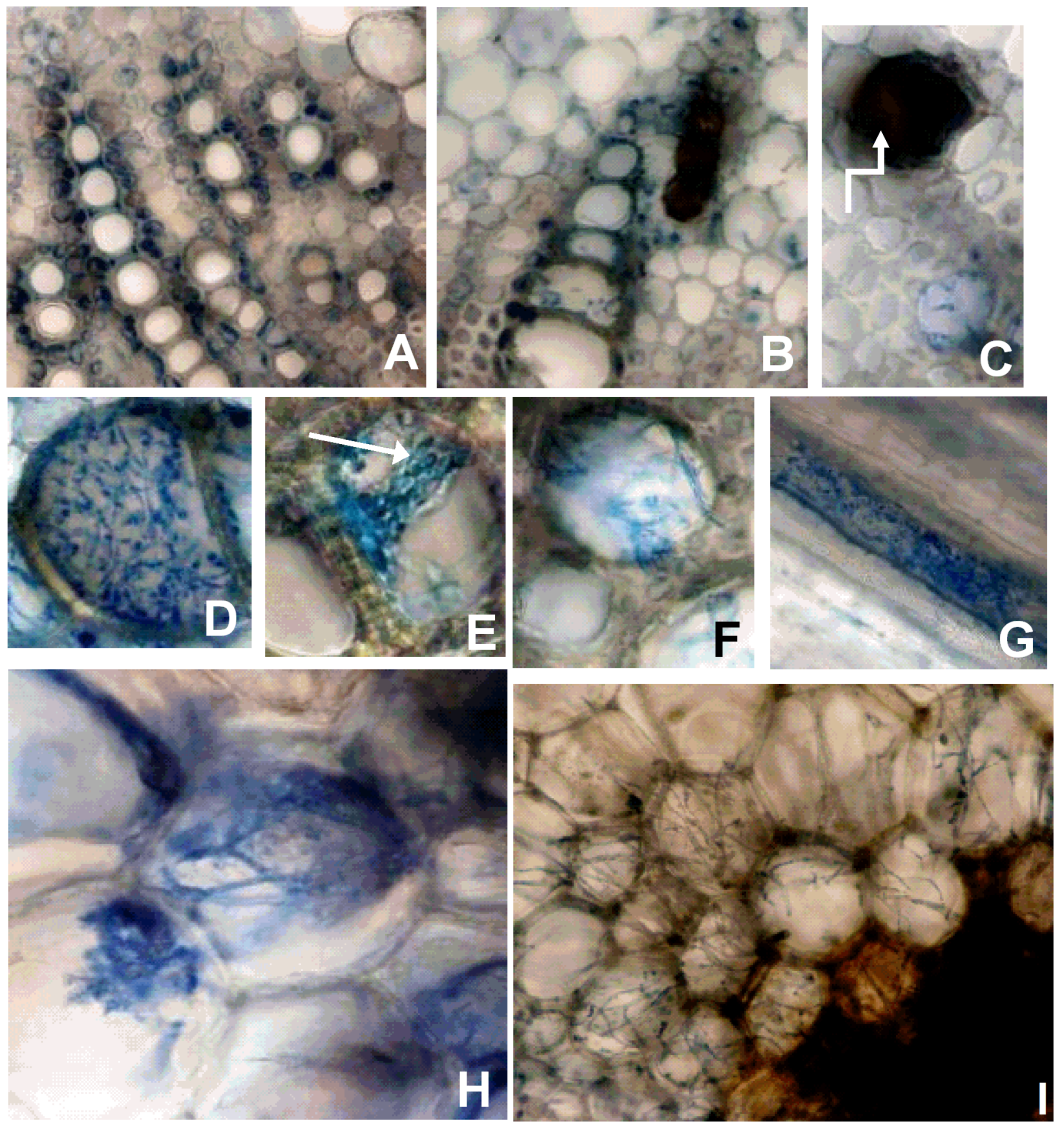
Pathogenic infection in soybean stems infected with *Phialophora gregata*. Pathogenic infection by wild-type isolates of types A and B of *Phialophora gregata* as observed 8 weeks post inoculation in cross sections of stems from a susceptible soybean cultivar (Corsoy 79). (A) No infection was observed in the non-inoculated control. (B) Inoculated with Type B. (C), A necrotic vessel with no fungal structures seen inside. (D), A vessel heavily colonized by *P. gregata*. (E)A vessel with evidence of sporulation (arrow). (F), Hyphae of *P. gregata* growing between vessels, possibly via pit pairs. (G), A longitudinal section of the xylem infected by *P. gregata*. (H and I), *P. gregata* beginning to colonize the parenchyma cells that compose the pith of the stem. Images A–G and I are 200x magnification, H 400x magnification.

Pathogenic infection was confirmed at 6 WPI based on observation of foliar and stem symptoms. Symptoms were observed 6 WPI to varying degrees in all inoculated cultivars, and there was a positive correlation between stem severity and the percent of vessels infected (R^2^ = 0.72) (Table 2). PgA and PgB were also detected in all cultivars using PCR and TLM analysis of stem tissues. Microscopic observations of tissue browning due to pathogenic infection at 6 to 8 WPI were similar in susceptible and resistant plants, although symptoms were more severe in susceptible plants. The fungus infected the xylem vessels in the middle and apex of resistant and susceptible cultivars with some vessels being 100% colonized by *P. gregata*, while others had severe browning but no fungal hyphae inside the vessel (Fig. 2B–D). At 8 WPI, hyphae were first observed moving into the adjacent parenchyma cells of the pith, and conidia were also observed in some vessels (Fig 2f–i).

**Figure 3:**
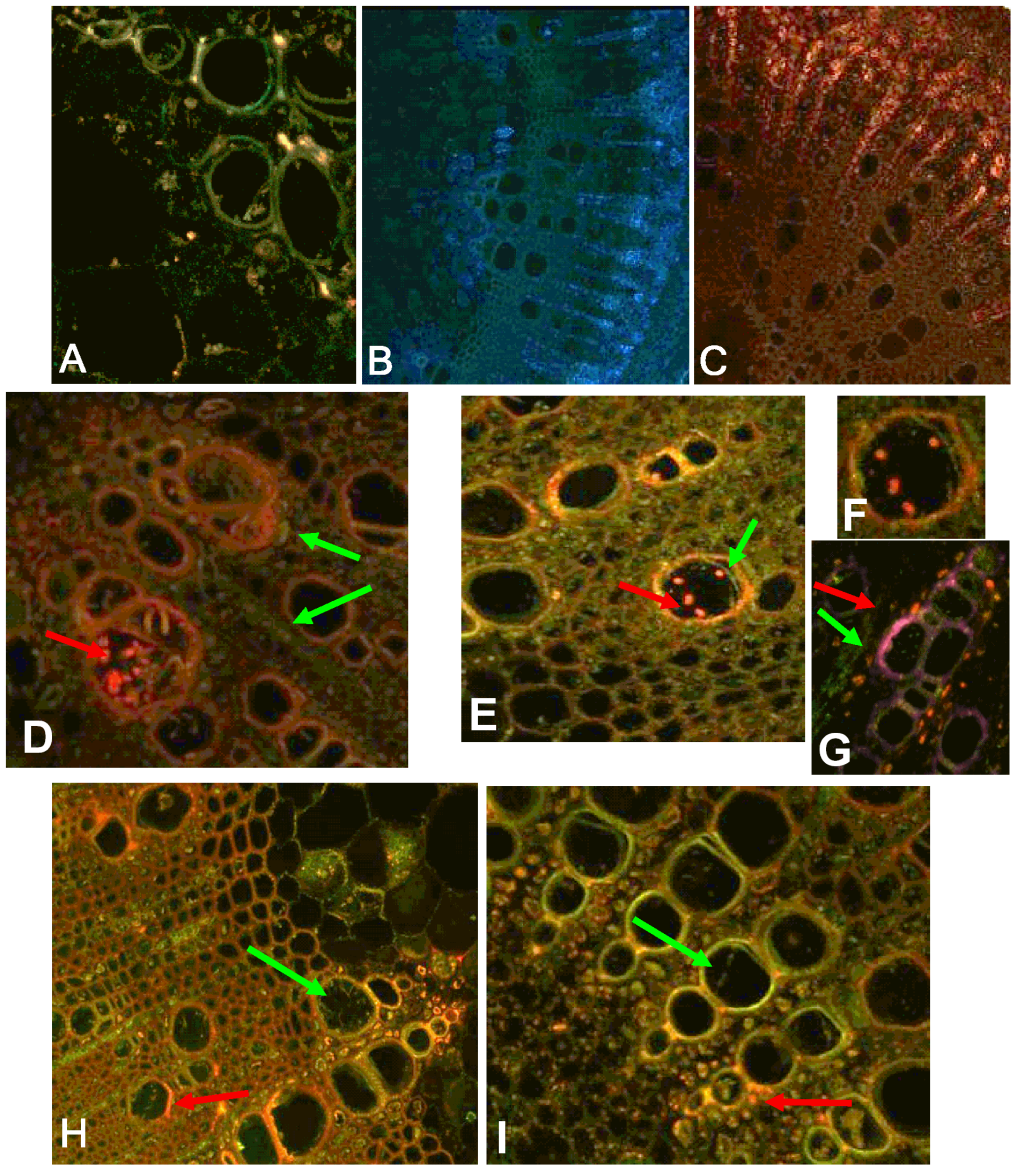
Latent and pathogenic infection in soybean stems infected with *Phialophora gregata* tagged with GFP or RFP. Latent (2 weeks post inoculation) and pathogenic infection (8 weeks post inoculation) of stems of Bell and Corsoy 79 either individually or co-inoculated with PgA-GFP or PgB-RFP. (A) PgA-GFP infecting a vessel of the resistant cultivar Bell during latent infection. No evidence of fungal infection was observed in the susceptible cultivar Corsoy 79 infected with either PgA-GFP (B) or PgB-RFP (C) during latent infection. (D) Bell co-inoculated with PgA-GFP (green arrow) and PgB-RFP (red arrow). (E and F) A xylem vessel of Bell colonized by both PgA-GFP and PgB-RFP. (F) is enlarged in for clarity. (G–I) Corsoy 79 inoculated with both types. A, D–I 200x magnification; B, C 100x magnification.

When PgA-GPF or PgB-RFP were separately inoculated in Bell or Corsoy 79, results were similar to those seen using the WT isolates and TLM. Neither type was observed in XS or LS during latent infection up to 3 WPI. As the interaction transitioned to pathogenic at 4 WPI, PgA-GPF and PgB-RFP infected the primary vessels, pith, and interfascicular region. When the two types were co-inoculated in plants, no differences were observed during latent infection, but differences in the infection pattern for each type was observed during pathogenic infection. Co-inoculation of Bell resulted in PgB-RFP being readily observed in the vessels, while PgA-GFP was limited to the interfascicular area outside of the primary and secondary vessel elements (Fig. 3). There were a few rare instances when both PgA-GFP and PgB-RFP could be observed in the same vessel (Fig. 3). The opposite infection patterns were observed in the susceptible cultivar. PgA-GFP readily colonized the primary vessels, while PgB-RFP was limited to the interfascicular region (Fig. 3). PgA-GFP and PgB-RFP were not seen co-infecting a vessel in the susceptible cultivar.

### Soybean anatomical responses to latent and pathogenic infection

Of the two separate experiments conducted using Bell and Corsoy 79, only data from experiment 1 is presented. Similar results were seen for experiment 2, unless noted otherwise.

**Figure 4:**
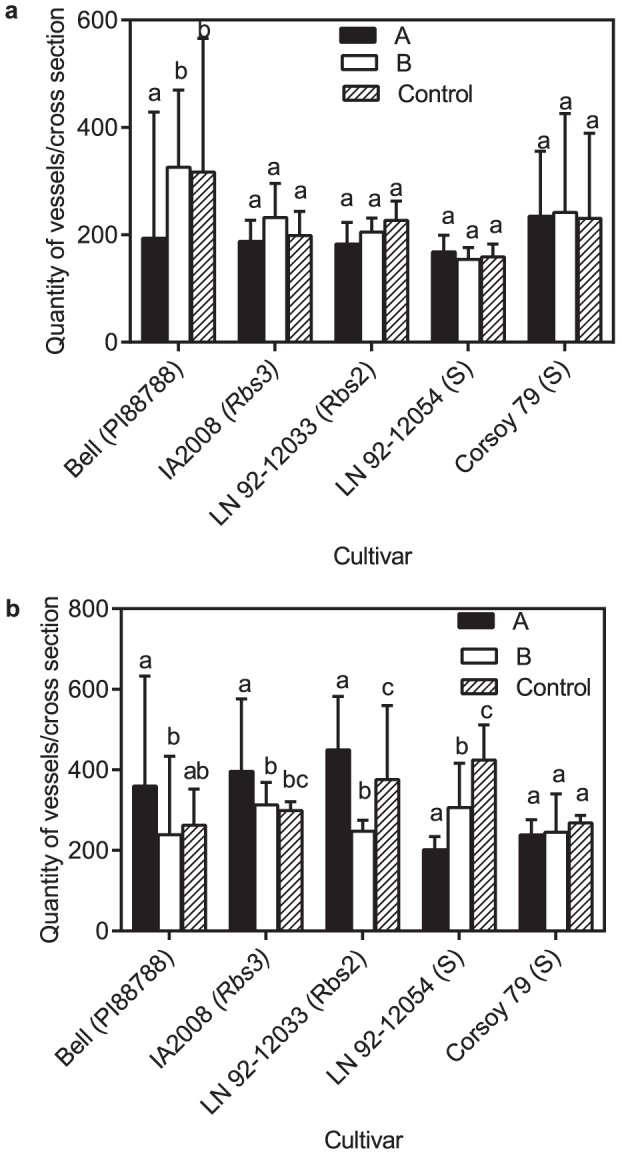
Average quantity of vessels of stems during latent and pathogenic infection with *Phialophora gregata*. Average quantity of vessels per cross section of stems two (A) and six (B) weeks post inoculation with either type A (PgA) or B (PgB) of *Phialophora gregata* for six soybean cultivars. Letters in parentheses next to cultivars indicate the source of BSR resistance or if the cultivar is susceptible (S). Treatments were compared using an ANOVA followed by a Fisher's LSD test, and bars with different letters are significantly different (*P* = 0.05). Error bars indicte 95% confidence intervals of the mean. Data shown is from experiment 1 and 3.

**Figure 5:**
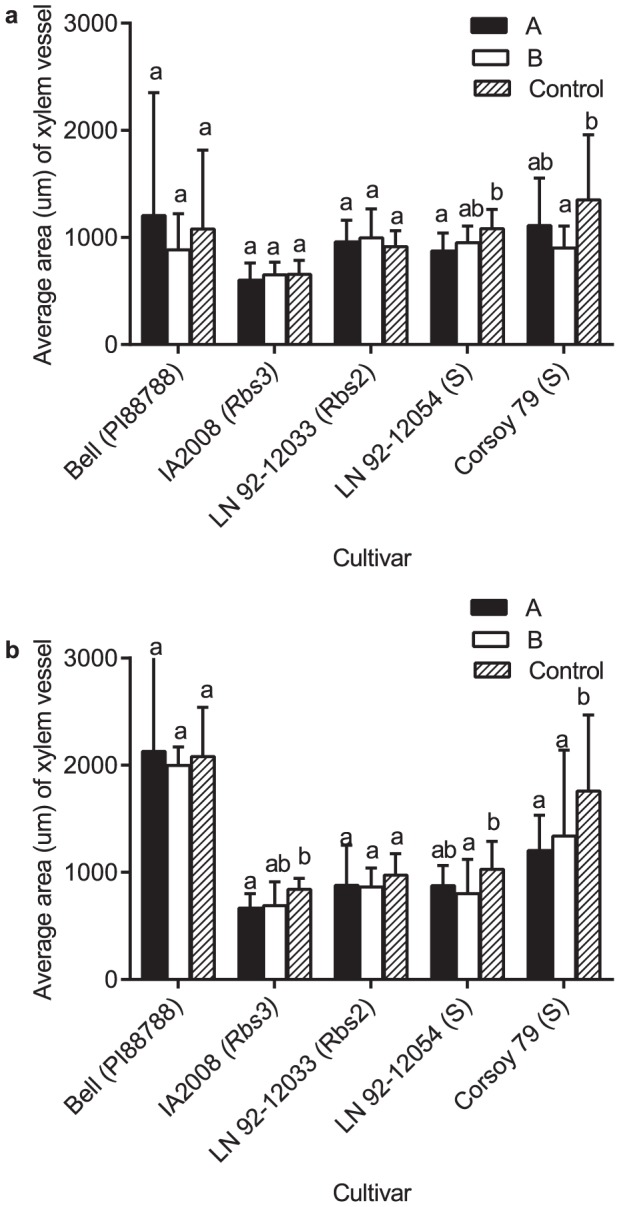
Average area of vessels of stems during latent and pathogenic infection with *Phialophora gregata*. Average area (um) of vessels of each cross section two (A) and six (B) weeks after inoculation with either type A (PgA) or B (PgB) of *Phialophora gregata* for six soybean cultivars. Letters in parentheses next to cultivars indicate the source of BSR resistance or if the cultivar is susceptible (S). Treatments were compared using an ANOVA followed by a Fisher's LSD test, and bars with different letters are significantly different (P = 0.05). Error bars indicate 95% confidence intervals of the mean. Data shown is from experiment 1 and 3. Treatments for experiment 1 were replicated three times (n = 3 plants/replication). Treatments for experiment 3 were replicated three times (n = 4 plants/replication).

At 2 WPI, differences in the quantity of vessels in infected and non-infected plants were not evident (Fig. 4A). The area of xylem vessels in resistant cultivars was not affected by infection, but the average area of vessels of the susceptible cultivars was reduced (Fig. 5A). The average vessel area of the susceptible cultivar, Corsoy 79, was significantly reduced when infected with types A and B when compared to the non-inoculated plants in experiment 1. These results were not significant during experiment 2, but a similar trend was observed. The average vessel area of the susceptible cultivar LN92-12054 was not reduced in plants infected with type A, but was reduced significantly when infected with type B compared to the control (Fig. 5A).

At 6 WPI, during pathogenic infection with PgA the resistant cultivars Bell, IA2008, and LN92-12033 had 20% more vessels compared to the uninfected plants (Table 2, Fig. 4B). During experiment 2, there was a trend for more vessels compared to the uninfected plants, but results were not significant (data not shown). No significant differences in vessel quantity were observed in Bell or IA2008 infected with PgB compared to the uninfected plants, but LN92-12033 had 30% fewer vessels compared to the non-infected control (data not shown). No significant differences in vessel quantity were observed in susceptible Corsoy 79 compared to the uninfected plants, but the susceptible cultivar LN 92-12054 had 50% fewer vessels compared to the uninfected plants (Fig. 4B). No significant differences in vessel quantity were observed in Corsoy 79 infected with PgB but LN92-12054 had 25% fewer vessels compared to the uninfected control.

The vessel area of the resistant plants infected with PgA or PgB compared to the uninfected plants was not significantly different during experiment at 6 WPI (Fig 5B). During experiment 1, the susceptible cultivar, Corsoy 79 had 25% less vessel area when inoculated with PgA and PgB compared to the control (Fig 5B). During experiment 2, the area of vessels was reduced when Corsoy 79 was inoculated with PgA and PgB, but results were only significant different from the control when inoculated with PgB. The susceptible cultivar LN92-12054 had reduced vessel area when inoculated with PgB but not PgA.

**Figure 6:**
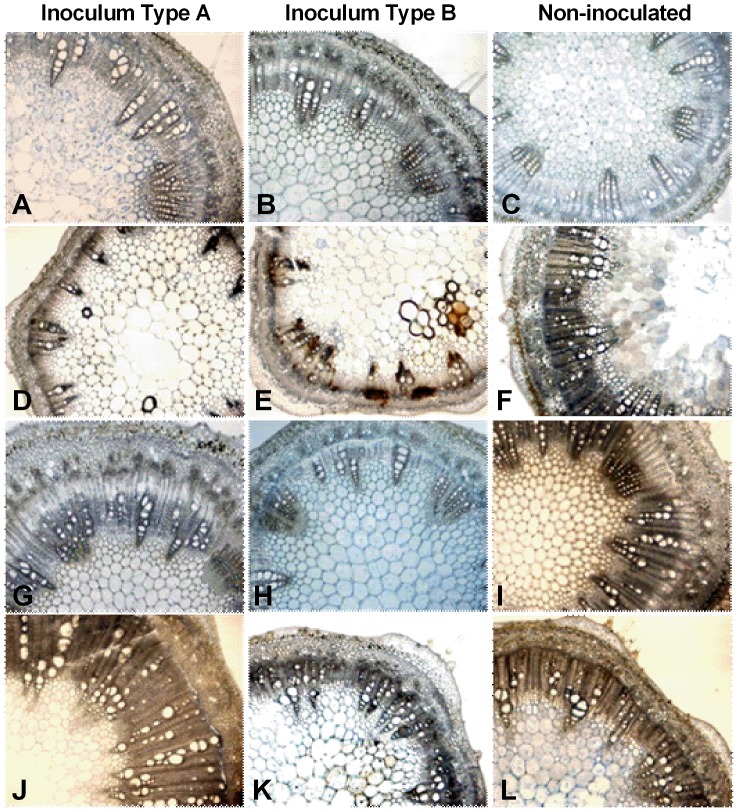
Stem anatomy of soybean during latent and pathogenic infection by *Phialophora gregata*. Stem anatomy of the resistant soybean cultivar (Bell) and the susceptible cultivar (Corsoy 79) during latent and pathogenic infection by types A and B of *Phialophora gregata*. During latent infection (2 weeks post inoculation) no differences were seen in anatomical organization in Corsoy 79 inoculated with type A (A), type B (B) or the control (C), but differences in the vascular cambium and xylem structure was seen during pathogenic infection (6 weeks post inoculation) (D–F). During latent infection of Bell, the vascular organization was similar in plants inoculated with type A (G) and the control (I), but plants infected with type B (H) had less secondary growth and a less pronounced layer of vascular cambium. Differences in anatomical organization were observed during pathogenic infection Bell inoculated with type B (K) had less secondary growth and less of a cambium layer compared to the Bell inoculated with type A (J) and the controls (F, L). All images were taken at 40x magnification. Treatments for experiment 1 were replicated three times (n = 3 plants/replication). Treatments for experiment 3 were replicated three times (n = 4 plants/replication).

During latent infection, typical soybean anatomy [21] was observed in both infected and non-infected plants. During pathogenic infection, the vascular tissue structure of infected plants differed from the uninfected plants. At 8 WPI, plants infected with *P. gregata* had the same anatomical features present, but to different extents. The width of the cylindrical ring of secondary xylem became smaller and less pronounced in infected plants compared to uninfected plants of Corsoy 79 (Fig. 6). A similar but less pronounced trend was observed in Bell. The secondary xylem of Bell was similar in plants infected with PgA and the uninfected plants (Fig. 6). Bell infected with PgB had less secondary xylem than the uninfected plants. Xylem vessels collapsed, the cambium layer began to breakdown, and vessel occlusions were also observed, but not to a great extent at 8 WPI when inoculated with either type A or B (Fig. 6).

## Discussion

Many pathogens are latent in plants for an extended period of time, however, little is known about infection patterns for pathogens during each stage or the effects of latent pathogenesis on plant growth. This study utilized a plant-pathogen system in which there are two genetically distinct types of the pathogen (*P. gregata*) that cause different symptoms in soybean. Our results are the first to identify differences in the vascular structure of resistant and susceptible cultivars during latent and pathogenic infection, provide insights into the mechanism of resistance to *P. gregata* and the active responses to infection, and reveal qualitative differences how the types interact and colonize when infecting plants.

The wild-type (WT) isolates of PgA and PgB were used to assess differences in internal stem anatomy of resistant and susceptible soybean cultivars during latent and pathogenic infection. During latent infection of resistant cultivars, neither the vessel quantity nor the average area of vessels was altered following infection with either type compared to the uninfected plants. Whereas in the two susceptible cultivars, the number of vessels was not affected, but the average area of vessels was reduced by 10 to 50% when infected with PgA and PgB compared to non-infected plants. Thus, the vascular anatomy of resistant plants was not altered during latent infection, while the susceptible plants had smaller vessels.

As the interaction between soybean and *P. gregata* became pathogenic, four main differences developed in the vascular system of resistant and susceptible plants. First, the resistant cultivars Bell, IA2008, and LN 92-12033, which are less susceptible to infection and colonization by type A than type B, had 20 to 25% more vessels when infected with type A or B than the uninfected plants [36], [37], [38]. Second, fewer than 10% of vessels of the resistant cultivars, Bell, IA2008, and LN 92-12033 were infected with type A, compared to greater than 50% of vessels of the susceptible cultivars Corsoy 79 and LN 92-12054. Third, the vessel area of plants was not an ideal method to separate resistant from susceptible cultivars, as the area appeared to be more dependent on cultivar genotype rather than on resistance or susceptibility. Fourth, the cambium layer, was smaller in susceptible plants infected with *P. gregata* compared to the uninfected plants, but was reduced to a lesser extent in infected resistant plants compared to uninfected.

These results suggest different mechanisms of resistance to *P. gregata* in soybean. One mechanism may be the regeneration of the vascular system following infection. The increase in vessel number and the unaltered cambium layer suggests resistant plants infected with type A may produce vessels to compensate for loss of vessel function and disrupted water movement through the plant due to vessel infection. Resistant plants infected with type B had vessel numbers similar to the non-inoculated controls. It was previously thought that type A was the more virulent pathogen, and most soybean breeding programs only evaluated for resistance to type A. Therefore, based on previous studies the resistant cultivars used in this study are thought to be resistant to type A and susceptible to type B [36], [37], [38]. Susceptible plants used in this study that were infected with type B had less secondary growth and a smaller cambium layer indicating the vascular tissues were unable to regenerate in response to infection by *P. gregata*. Similarly, reduced vessel number, diameter, and vascular cambium were observed in tomato (*Lycopersicon esculentum*) infected with *Fusarium oxysporum* f. sp. *lycopersici* compared to the uninfected plants [12]. Another potential resistance mechanism is the ability of resistant plants to restrict or exclude the pathogen from the vascular system. Less than 10% of vessels of the resistant cultivars, Bell, IA2008, and LN 92-12033 were infected with type A, compared to greater than 50% of vessels of the susceptible cultivars LN 92-12054 and Corsoy 79. Resistant cultivars infected with type B had 6–30% of vessels infected and susceptible 15–30%. These results are similar to some other plant-pathogen systems during pathogenic infection in that resistant cultivars are able to restrict pathogen growth [13], [39].

Latent and pathogenic infection were distinguished by differences in infection patterns. During latent infection, fungal infection was rarely observed in any plant tissues using either transmitted or fluorescent light microscopy, however, the fungus was detected in some but not all stems of plants using sPCR and/or qPCR at 1 or 2 WPI. We hypothesize there were only trace amounts of *P. gregata* within the stem at this time, thus making it difficult to visualize within the plants. *P. gregata* may also have been surviving intercellularly as do many other asymptomatic fungal colonizers [7], [10], [40]. Alternatively, *P. gregata* may have been limited to the roots during latent infection. Other studies in different plant-pathogen systems have shown that some vascular pathogens are limited to the root due to vascular occlusions [39], [41],[42]. However, our limited observations of roots suggest this was not the case since *P. gregata* was rarely observed in the roots. We are confident that *P. gregata* was in the stems based on our PCR results and because *P. gregata* can be isolated from the apex of infected plants one hour post-inoculation, suggesting conidia move throughout the vascular system and systemically colonize the plant during latent infection [22], [43]. A single conidium is 3.4×7.6 µm and is smaller than the average soybean vessel. From our results, we concur with Tabor et al. (2007) that the fungus is capable of moving to the apex of plants during the early stages of infection, but the amount of fungal material is minimal.

During pathogenic infection, *P. gregata* was readily observed in plant stems using both TLM and fluorescent LSCM. By 4 WPI, type A and type B were present in the vascular system of stems in resistant and susceptible cultivars and vascular browning was observed microscopically, but macroscopic symptoms were not observed. By 6 WPI, macroscopic symptoms of tissue damage were observed in the stem and on the leaves, and the two pathogen types had begun to colonize the primary vessels of the vascular system. Fungal hyphae were observed in areas with and without evidence of stem browning. Vascular infection was required before stem browning and before infection of the pith tissues was observed.

Transformation of type B of *P. gregata* to express RFP was successful and was useful to clarify infection and infection patterns by the two pathogen types. The PgB-RFP isolate behaved like the WT isolate in all aspects tested. The fluorescent isolates confirmed the observations of the WT isolates using TLM and also offered novel insight into differences in pathogenic infection of soybean by the PgA-GFP and PgB-RFP isolates. PgA-GFP infected vessels of Corsoy 79, but PgB-RFP was limited to the interfascicular areas during pathogenic infection of co-inoculated plants. Corsoy 79 is susceptible to both types so our data suggests that PgA is a better colonizer of the vascular system than PgB when the two types are co-inoculated. The xylem is an ideal microhabitat for many pathogens due to the presence of water and nutrients, and because propagules can be transported systemically throughout the plant. In Bell, which is susceptible to PgB and resistant to PgA, PgA-GFP was limited to the interfascicular region while PgB-RFP was observed in the primary vessels [19], [37].

Latent infection is a well-known but poorly understood phenomenon that is often considered one of the highest forms of parasitism since plant and pathogen can co-exist, in some cases, for long periods of time [2], [4]. Our results indicate that during latent infection, pathogens are excluded from intracellular spaces, and soybean anatomy does not differ compared to uninfected plants. During pathogenic infection, pathogens can be found intracellularly and anatomy of plants can be modified to compensate for infection. More specifically, our results suggest susceptibility to *P. gregata* is a result of the pathogen colonizing the vascular system, while resistant plants are able to exclude it to the interfascicular region and produce more vessels. Many questions remain on differences in latent and pathogenic infection in this and other pathosystems. It is unknown what initiates the pathogen to move into the vascular system, and how resistant plants restrict infection of vessels. Future studies should investigate if type A is more aggressive or is a better competitor and grows faster than type B and if latent infection has a significant negative impact on the host.
